# OVERWEIGHT, PERCEIVED ENVIRONMENT, AND SOCIAL DEPRIVATION: A STUDY ON THE PERCEPTION OF PARENTS OR GUARDIANS

**DOI:** 10.1590/1984-0462/;2018;36;4;00011

**Published:** 2018

**Authors:** Maíra Macário de Assis, Maria Alvim Leite, Alessandra Jordão Côrtes, Ariene Silva do Carmo, Fernanda Penido Matozinhos, Ana Paula Carlos Cândido, Larissa Loures Mendes

**Affiliations:** a Universidade Federal de Juiz de Fora, Juiz de Fora, MG, Brasil.; b Universidade de São Paulo, São Paulo, SP, Brasil.; c Universidade Federal de Minas Gerais, Belo Horizonte, MG, Brasil.

**Keywords:** Socioeconomic factors, Feeding, Leisure activities, Fatores socioeconômicos, Alimentação, Atividades de lazer

## Abstract

**Objective:**

To investigate parents’ or guardians’ perception of their residential proximity to food retailers, leisure areas, and spaces for physical activity according to neighborhood social deprivation, and test associations between the perceived environment and their children’s overweight.

**Methods:**

Cross-sectional study conducted with 408 children and adolescents (6- to 15-year-olds) attending public schools in a medium-sized Brazilian city. Data were collected from 2011 to 2014. A telephone interview using a structured research tool determined the presence of overweight and the walking time between the participants’ home and the places evaluated. The indicator of social deprivation adopted was the Health Vulnerability Index. Logistic regression models were constructed to predict the perception of proximity (social deprivation as an explanatory variable) and evaluate perceived environmental factors (explanatory variables) associated with overweight (outcome).

**Results:**

Residents of areas with higher social vulnerability showed a probability of perceived proximity 50 to 71% lower to supermarkets, street/produce markets, parks, recreation areas/community centers, and gyms compared to residents of less vulnerable areas. The perceived proximity to parks reduced the chance of overweight in children and adolescents in 73%, with an odds ratio (OR) of 0.27 (95%CI 0.07-0.95; p<0.05).

**Conclusions:**

The perceived environment of the residential area infrastructure might be related to neighborhood social deprivation and the presence of overweight in children and adolescents.

## RESUMO

**Objetivo:**

Investigar a percepção de pais ou responsáveis sobre a proximidade residencial a locais de venda de alimentos, de lazer e de atividade física segundo a privação social da vizinhança e testar associações do ambiente percebido com o excesso de peso de seus filhos.

**Métodos:**

Estudo transversal conduzido com 408 crianças e adolescentes (6 a 15 anos de idade) de escolas públicas de um município de médio porte brasileiro. A coleta de dados ocorreu de 2011 a 2014. Foram determinados a presença de excesso de peso e o tempo de deslocamento da residência dos participantes aos locais avaliados, questionados durante entrevista telefônica com instrumento de pesquisa estruturado. O indicador de privação social adotado foi o Índice de Vulnerabilidade da Saúde. Modelos de regressão logística foram construídos para predizer a proximidade percebida (privação social como variável explicativa) e para avaliar os fatores do ambiente percebido (variáveis explicativas) associados ao excesso de peso (desfecho).

**Resultados:**

Moradores de áreas com maior vulnerabilidade social apresentaram chances de percepção de proximidade 50 a 71% menores para supermercados, feiras/sacolões, parques, áreas de lazer/centros comunitários e academias de ginástica, quando comparados a residentes de áreas com menor vulnerabilidade. A percepção de proximidade a parques reduziu em 73% a chance de excesso de peso em crianças e adolescentes, com *Odds Ratio* (OR) de 0,27 (IC95% 0,07-0,95; p<0,05).

**Conclusões:**

A percepção ambiental sobre a infraestrutura da área residencial pode estar relacionada com a privação social da vizinhança e a presença de excesso de peso em crianças e adolescentes.

## INTRODUCTION

Obesity in children and adolescents is a public health issue in several countries due to its progressive prevalence increase in recent decades, and for being an important risk factor for the development of chronic non-communicable diseases, which contribute to reduce life quality and expectancy.^1^ As a multifactorial disease, besides genetic components and lifestyle habits, family, social, and environmental contexts can be predictors of weight gain.^2^

Regarding the influence of the environment on food consumption, in developed countries, the lower distance and higher density of fast food restaurants and convenience stores in the residential surroundings are associated with more monotonous and highly caloric food choices among children and adolescents.^3^ Moreover, the lack of leisure spaces and facilities can hinder the practice of physical activity.^1^ Together, these factors represent an obesogenic behavior and contribute to the increase in obesity prevalence for this age group.^4^

The social environment includes different aspects, such as housing, safety, income, and access to healthcare services, which can also influence the behavior of individuals and health outcomes, be it by creating opportunities and facilitating the decision-making process, or by hindering them.^5^ The concept of social deprivation has been used to evaluate this environment with the purpose of identifying, measuring, and explaining the forms of inequality based on socioeconomic conditions.^6^ In this scenario, neighborhoods with greater social deprivation are the most affected, presenting less diversity of trades and services, which is possibly a result of the scarcity of local resources and infrastructure, and high crime rates.^7^

International data on the availability and access to food retailers showed that children and adolescents who live close to stores specialized in the sale of fresh and unprocessed foods - such as produce markets -,^8^ away from places that sell unhealthy foods - e.g., fast food restaurants -,^9^ and in areas with a higher number of supermarkets that predominantly sell healthy foods over unhealthy ones^10^ showed lower obesity prevalence.

In Brazil, studies on the environment focused on objective measures suggest that residents of higher income neighborhoods have better access to all types of food retailers, including supermarkets and street markets.^11^ However, studies that investigate parents’ or guardians’ perception of environmental aspects and relate them to the nutritional status of their children are still scarce^12^, and, currently, there are none that consider the contrasts between the different socioeconomic status of the place of residence.

Considering the importance of exploring the environmental inequalities that could affect health conditions, the purpose of the present study was to investigate the differences in parents’ or guardians’ perception of their residential proximity to food retailers and leisure areas/spaces for physical activity in levels of neighborhood social deprivation. We also aimed to test the associations between the perceived environment and their children’s overweight.

## METHOD

This is a cross-sectional study conducted with children and adolescents aged 6 to 15 years attending public schools and their parents or guardians in a medium-sized Brazilian city (Juiz de Fora, Minas Gerais). Data were collected from 2011 to 2014.

According to the 2009 School Census,^13^ the number of children and adolescents enrolled in public schools in Juiz de Fora was 71,671. To calculate the sample size, we used an estimate of proportion of 50% for a given characteristic, a value that provides the largest sample size for a finite population (71,671), and set the significance level (alpha or type I error) and sampling error in 5%.^14^ ^,^ ^15^ Thus, the estimated n sample comprised 383 participants.

The sample design was probabilistic and clustered in three stages:

1. schools - randomly and proportionally selected in each of the seven administrative districts of the city, totaling 36 schools;
2. classes - all classes by grade that met the age group of interest were included;
3. students - randomly and proportionally selected in each grade, totaling 708 students.

We considered this number due to possible losses caused by absences on the day of data collection or parents who refused to let their children participate. Weighting factors were not used in the sample design since the purpose of the study was not to extrapolate the findings to the population of children and adolescents attending public schools in the city.

The first step was to collect anthropometric measurements of weight and height of the students, which happened in a private room in the schools. Trained researchers conducted these measurements, using a calibrated digital scale (weight in kg) and a portable stadiometer (height in m). The cut-off point for overweight was z score>+1 standard deviation of body mass index (BMI) for age, according to the growth curves of the World Health Organization (WHO).^16^

In addition, a questionnaire prepared by the research team of the present study was used to collect socioeconomic and demographic data (available with the authors). The participants answered questions about age, gender (male or female), ethnicity/skin color (white, multiracial, or black), years of schooling of the parent or guardian (less or more than 11 years of study), and household monthly income (collected in Brazilian reals and categorized into quartiles of distribution). The multiracial category included children and adolescents who reported having light to dark brown skin color. The category of more than or equal to 11 years of study included parents or guardians with the following levels of education: complete high school, complete and incomplete higher education; for the category of less than 11 years of study, the levels were: illiterate, complete and incomplete elementary school, complete and incomplete middle school, and incomplete high school.

**Figure 1:**
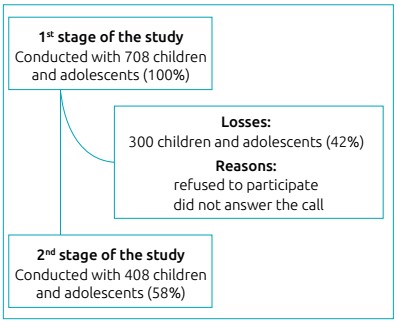
Flowchart of the sample composition.

To collect information on the perceived food environment of the residential neighborhood of children and adolescents, a subsample of 408 children and adolescents (58% of the initial sample) and their parents or guardians agreed to participate (Figure 1). The information was collected via telephone call, in which parents and/or guardians of the participants answered an adapted selection of the validated Portuguese version of the Neighborhood Environment Walkability Scale (NEWS),^17^ an instrument that evaluates perceived environmental characteristics. The full scale has questions about the perception of access to services, the existence of sidewalks and bikeways, traffic safety, and security against crimes among others, represented by two categories of answer - “yes” and “no” or “agree” and “disagree.” It also includes questions on the perception of walking time to different types of establishments in the neighborhood of residence.

The questions selected from the scale for this study were related to the proximity to food retailers and leisure areas/spaces for physical activity. More specifically, those associated with mini-market, supermarket, street/produce market, park, recreation area/community center, and gym; and the walking time (or proximity) from the family’s residence to these sites (up to 10 minutes, 11 to 20 minutes, more than 20 minutes, and does not have). The category “does not have” refers to the lack of perception of food retailers or leisure areas/spaces for physical activity in the neighborhood. Recreation area was defined as a public area, without buildings, intended for active games.^18^ For the walking time/proximity to food retailers variable, we assumed that, on average, an adult tends to walk 400 m in 5 minutes, that is, almost 500 m, and adolescents, at a moderate pace, are able to walk more than 1,500 m in 15 minutes.^2^

The indicator of social deprivation used was the Health Vulnerability Index (HVI).^19^ To construct it, we used information from the 2010 Census.^20^ This indicator was dichotomized into low vulnerability (low or medium risk) and high vulnerability (high or very high risk). The synthetic index takes into account sanitation and socioeconomic aspects (such as water supply, sanitary sewer, waste destination, residents per household, illiteracy, income, and ethnicity/skin color) in its construction in order to analyze the characteristics of population groups in certain geographical areas and reveal the inequalities in the epidemiological profile of different social groups.^19^

The statistical analysis included the calculation of relative and absolute frequency distribution. To test the association between sociodemographic characteristics and overweight among children and adolescents, we used the chi-square test. Simple multinomial logistic regression models were constructed to predict the perceived proximity to food retailers, leisure areas, and spaces for physical activity. All models used the HVI classification of the residence as an explanatory variable. Odds ratio (OR) with a confidence interval of 95% (95%CI) was used as an effect measure. To evaluate perceived environmental factors associated with overweight, we used simple and multiple binary logistic regressions, with overweight as outcome, and aspects of the perceived environment (perceived proximity to food retailers, leisure areas, and spaces for physical activity) as explanatory variables. These models were adjusted for gender and age of children or adolescents, schooling of parents or guardians, monthly household income, and HVI. We used the Hosmer-Lemeshow test to verify the adjustment of the multiple model. OR with 95%CI was used as an effect measure. All analyses adopted a significance level of 5% (p<0.05).

The Committee for Ethics in Research of Universidade Federal de Juiz de Fora (CEP/UFJF) approved this project, according to the terms defined in Resolution No. 466/12 of the National Health Council, Report No. 522,694.

## RESULTS

408 children and adolescents aged 6 to 15 years participated in the study. Out of them, 53.4% (n=218) were female, and 74.85% (n=305) were adolescents. The overweight prevalence was 34.3% (n=140). Most participants declared being multiracial (68.4%; n=264) and lived in less vulnerable areas according to their HVI (59.8%; n=244). Regarding parents or guardians, 51.6% (n=160) of them reported having 11 or more years of study, and monthly household income ranged from R$ 400.00 to R$ 8,500.00.

Table 1 presents the socioeconomic and demographic characteristics of children and adolescents stratified by overweight presence. There were no statistically significant differences.

**Table 1:** Descriptive statistics of socioeconomic and demographic characteristics of children and adolescents according to overweight presence. Juiz de Fora, Minas Gerais, 2011-2014.

Characteristic	Total	Overweight				p-value
		Yes		No		
	n	%	n	%	n	
Age group (years)						
6 - 9	103	36.9	38	63.1	65	0.524
10 - 15	305	33.4	102	66.6	203	
Gender						
Female	218	30.7	67	69.3	151	0.103
Male	190	38.4	73	61.6	117	
Ethnicity/skin color						
White	83	26.5	22	73.5	61	0.153
Multiracial	264	37.1	98	62.9	166	
Black	39	41.0	16	59.0	23	
Health Vulnerability Index						
Low vulnerability	244	34.0	83	66.0	161	0.877
High vulnerability	164	34.8	57	65.3	107	
Schooling of the parent or guardian						
Illiterate/1 to 3 years of study	31	29.0	9	71.0	22	0.329
4 to 10 years of study	119	42.0	50	58.0	69	
11 or more years of study	160	35.6	57	64.4	103	
Household monthly income						
1º quartile (R$ 400.00 to R$ 933.00)	79	29.1	23	70.9	56	0.615
2º quartile (R$ 934.00 to R$ 1,300.00)	83	37.3	31	62.7	52	
3º quartile (R$ 1.301.00 to R$ 2,005.00)	70	38. 6	27	61.4	43	
4º quartile (R$ 2.006.00 to R$ 8,500.00)	77	35.1	27	64.9	50	

Regarding food retailers, compared to residents of less vulnerable neighborhoods, those who live in areas with higher social vulnerability showed a probability of perceived proximity 55 and 60% lower to supermarkets up to 10 minutes away (OR 0.45; 95%CI 0.25-0.80) and 11 to 20 minutes away (OR 0.40; 95%CI 0.22-0.75), respectively; and 50 and 51% lower to street/produce markets up to 10 minutes away (OR 0.50; 95%CI 0.27-0.90) and 11 to 20 minutes away (OR 0.51; 95%CI 0.26-0.97), respectively (Table 2). With respect to leisure areas/spaces for physical activity, regions of higher vulnerability had 66% less chance of perceived proximity to parks 11 to 20 minutes away (OR 0.34; 95%CI 0.12-0.94), and 50% for recreation areas/community centers up to 10 minutes away (OR 0.50; 95%CI 0.29-0.85). Still on residents of more vulnerable areas, the probability of perceived proximity to gyms was 68, 71, and 66% lower for distances up to 10 minutes (OR 0.32; 95%CI 0.19-0.52), 11 to 20 minutes (OR 0.29; 95%CI 0.16-0.52), and more than 20 minutes (OR 0.34; 95%CI 0.17-0.68), respectively (Table 2).

**Table 2:** Simple multinomial logistic regression analyses to predict the perceived proximity to different food retailers and leisure areas/spaces for physical activity based on the Health Vulnerability Index of the residence. Juiz de Fora, Minas Gerais, 2011-2014.

Characteristic	Total % (n)	Vulnerability		OR (95%CI)	p-value^a^
		Low % (n)	High % (n)		
Food retailers					
Mini-market					
Does not have	6.86 (28)	8.20 (20)	4.88 (8)	Reference	-
Up to 10 min	3.68 (15)	2.87 (7)	4.88 (8)	0.53 (0.18-1.52)	0.243
11 to 20 min	67.65 (276)	70.08 (171)	64.02 (105)	0.81 (0.27-2.44)	0.719
More than 20 min	21.81 (89)	18.85 (46)	26.22 (43)	0.35 (0.09-1.28)	0.155
Supermarket					
Does not have	21.81 (89)	18.85 (46)	26.22 (43)	Reference	-
Up to 10 min	18.38 (75)	14.34 (35)	24.39 (40)	0.45 (0.25-0.80)	0.007
11 to 20 min	34.31 (140)	37.70 (92)	29.27 (48)	0.40 (0.22-0.75)	0.004
More than 20 min	25.49 (104)	29.10 (71)	20.12 (33)	0.82 (0.44-1.51)	0.522
Street/produce market					
Does not have	12.04 (49)	11.93 (29)	12.20 (20)	Reference	-
Up to 10 min	14.50 (59)	11.11 (27)	19.51 (32)	0.50 (0.27-0.90)	0.022
11 to 20 min	47.42 (193)	49.79 (121)	43.90 (72)	0.51 (0.26-0.97)	0.042
More than 20 min	26.04 (106)	27.16 (66)	24.39 (40)	0.58 (0.27-1.25)	0.166
Leisure areas/spaces for physical activity					
Park					
Does not have	2.45 (10)	3.28 (8)	1.22 (2)	Reference	-
Up to 10 min	85.54 (349)	81.15 (198)	92.07 (151)	0.41 (0.16-1.06)	0.067
11 to 20 min	6.13 (25)	7.79 (19)	3.66 (6)	0.34 (0.12-0.94)	0.038
More than 20 min	5.88 (24)	7.79 (19)	3.05 (5)	0.32 (0.06-1.56)	0.162
Recreation area/community center					
Does not have	5.93 (24)	6.20 (15)	5.52 (9)	Reference	-
Up to 10 min	57.53 (233)	52.89 (128)	64.42 (105)	0.50 (0.29-0.85)	0.010
11 to 20 min	21.98 (89)	26.03 (63)	15.95 (26)	0.77 (0.43-1.39)	0.401
More than 20 min	14.57 (59)	14.88 (36)	14.11 (23)	0.73 (0.30-1.73)	0.479
Gym					
Does not have	11.55 (47)	24.28 (59)	9.15 (15)	Reference	-
Up to 10 min	36.61 (149)	25.93 (63)	52.44 (86)	0.32 (0.19-0.52)	<0.001
11 to 20 min	31.45 (128)	13.17 (32)	23.78 (39)	0.29 (0.16-0.52)	<0.001
More than 20 min	20.39 (83)	36.63 (89)	14.63 (24)	0.34 (0.17-0.68)	0.003

Min: minutes; OR: odds ratio; 95%CI: confidence interval of 95%; p-value<0.05; ^a^in all models, the explanatory variable was the Health Vulnerability Index (0: low vulnerability; 1: high vulnerability).

Table 3 describes simple and multiple binary logistic regression analyses to predict overweight among the children and adolescents evaluated. The simple analysis, when adjusted for potential confounding factors, showed that parents’ or guardians’ perceived proximity to parks (up to 10 minutes) reduced in 73% the chances of overweight in children and adolescents (OR 0.27; 95%CI 0.07-0.95). This association remained significant even after adjustment for other independent variables (OR 0.21; 95%CI 0.06-0.81). The other variables were not associated with overweight.

**Table 3:** Simple and multiple logistic regression analyses to predict overweight among children and adolescents. Juiz de Fora, Minas Gerais, 2011-2014.

Characteristic	OR^a^	95%CI	p-value	OR^b^	95%CI	p-value^b^
Food retailers						
Mini-market						
Does not have	1.00	-		1.00	-	
Up to 10 min	3.40	0.39-29.68	0.269	2.87	0.28-29.69	0.377
11 to 20 min	2.92	0.32-26.69	0.343	3.50	0.32-38.64	0.306
More than 20 min	3.23	0.32-32.43	0.320	3.67	0.29-45.68	0.312
Supermarket						
Does not have	1.00	-		1.00	-	
Up to 10 min	1.94	0.92-4.06	0.080	1.47	0.60-3.56	0.398
11 to 20 min	1.69	0.76-3.77	0.199	1.58	0.64-3.92	0.324
More than 20 min	1.59	0.70-3.62	0.265	1.38	0.54-3.49	0.499
Street/produce market						
Does not have	1.00	-		1.00	-	
Up to 10 min	1.85	0.87-3.94	0.108	1.80	0.75-4.34	0.188
11 to 20 min	0.89	0.38-2.08	0.792	0.76	0.30-1.92	0.564
More than 20 min	1.43	0.53-3.82	0.477	1.31	0.41-4.20	0.649
Leisure areas/spaces for physical activity						
Park						
Does not have	1.00	-		1.00	-	
Up to 10 min	0.27	0.07-0.95	0.041	0.21	0.06-0.81	0.024
11 to 20 min	1.82	0.70-4.77	0.217	1.54	0.53-4.45	0.429
More than 20 min	0.20	0.02-1.67	0.137	0.15	0.01-1.50	0.106
Recreation area/community center						
Does not have	1.00	-		1.00	-	
Up to 10 min	1.41	0.75-2.64	0.281	1.67	0.84-3.32	0.145
11 to 20 min	1.57	0.78-3.17	0.206	1.48	0.67-3.29	0.337
More than 20 min	1.34	0.48-3.73	0.575	2.57	0.70-9.45	0.156
Gym						
Does not have	1.00	-		1.00	-	
Up to 10 min	1.05	0.58-1.93	0.862	0.71	0.35-1.40	0.321
11 to 20 min	1.25	0.62-2.53	0.532	1.02	0.46-2.25	0.963
More than 20 min	0.63	0.27-1.47	0.285	0.45	0.17-1.23	0.119

Min: minutes; OR: odds ratio; 95%CI: confidence interval of 95%; p-value<0.05; ^a^simple regression adjusted for gender and age of the child or adolescent, schooling of the parent or guardian, household monthly income, and Health Vulnerability Index; ^b^multiple regression adjusted for gender and age of the child or adolescent, schooling of the parent or guardian, household monthly income, and Health Vulnerability Index. Model adjustment: Goodness of fit = 0.121.

## DISCUSSION

The results showed that the perceived proximity to food retailers and leisure areas/spaces for physical activity varied according to neighborhood social deprivation, with residents of less vulnerable areas having the perception of being closer to supermarkets, street/produce markets, parks, recreation areas/community centers, and gyms. Moreover, they suggest that the reported proximity to parks acted as a protective factor for overweight in children and adolescents.

This study did not evaluate the aspects of the built environment directly, but through the subjective perception of individuals, which tends to be closer to the actual characteristics of the physical environment^21^ and are important, as people take into account their perception of the environment where they live when making decisions.^5^ In the group of children and adolescents, family members are determinant to food choices and lifestyle. Also, according to the literature, parents’ or guardians’ negative perception of food environment characteristics was associated with lower availability of fruits at home^22^ ^,^ ^23^, and their perceived proximity to leisure areas and spaces for physical activity was positively correlated to their children’s recreation and physical activity time (reported by them).^23^

The present study revealed variation in reported walking time, according to the level of neighborhood social deprivation. This scenario can be explained by the better infrastructure of trades and services, such as food retailers, which tend to be present in more affluent areas,^24^ in addition to the establishment of parks and public places for the practice of physical activity and better transport system.^11^ These factors can affect the lifestyle and eating habits of families, as the presence of parks, sports facilities, and healthy food retailers near the residence might encourage the practice of physical activity and appropriate food consumption, with direct implications on health.^1^

On the other hand, areas with higher socioeconomic vulnerability had a lower probability of perceived proximity to all types of food retailers. This shortage of services could be due to the insecurity caused by higher crime rates, the precarious urban infrastructure, and the low socioeconomic level of the inhabitants of these areas, which limits their purchasing power^25^ and, in turn, makes these regions less attractive to entrepreneurs, discouraging the establishment of stores in these surroundings.^7^ Such social disadvantages contribute to create obesogenic environments that put children and adolescents at greater risk of gaining weight.^1^

Regarding the association between parents’ or guardians’ perceived proximity to parks and a lower chance of overweight among children and adolescents, recent studies have investigated the influence of perceived environment on nutritional status.^23^ ^,^ ^26^ In Itirapuã, a small city in São Paulo, living further from public squares or outdoor areas where it is possible to practice physical activities increased the chance of overweight in adults (OR 2.05; 95%CI 1.15-3.66).^26^

A North American study conducted with children and adolescents aged 10 to 17 years found that those who lived in unfavorable social conditions, such as unsafe environment, poor houses, and without access to sidewalks, parks, and recreation centers, had 20 to 60% more chance of being obese or overweight.^4^

In cities, parks are public spaces intended not only for walking, commuting, practicing physical activity, and recreation but also as a place suited for social interaction, especially for the part of the population without access to private environments for physical activity, such as clubs and gyms.^27^ Thus, measures such as proper maintenance, safety, and easy access can encourage their use. For instance, parents could allow their children to play outside, reducing the time spent on computer activities or watching television, reinforcing an active lifestyle.^27^ ^,^ ^28^

Some limitations of the present study are: first, the cross-sectional design, which reveals associations without evaluating causalities. Also, the study did not assess the practice of physical activity and food consumption, factors directly related to nutritional status. Only public-school students were evaluated, so the findings should not be extrapolated to all children and adolescents in the city. Nonetheless, considering that many of the public policies outlined also influence this group, it is crucial to study this population. The questionnaire with information about the proximity to food retailers, leisure areas, and spaces for physical activity was answered via telephone call, based on parents’ or guardians’ reports of their perceived environment, rather than direct observation. However, other similar studies conducted via telephone interview^29^ confirmed the validity and reproducibility of the NEWS questionnaire,^17^ and identified the existence of associations between perceived and reported environmental characteristics and those measured in a direct way.^21^ It is also important to clarify that the study did not assess which food retailers families used the most, only their residential proximity.

In view of the above, it is relevant to investigate perceived environmental aspects, given their importance in the decision-making process, and consider the social and infrastructure elements related to healthy eating habits, leisure, and the practice of physical activities in urban planning to make the creation of environments that promote health possible.
